# Global Research Trends on Monkeypox Virus: A Bibliometric and Visualized Study

**DOI:** 10.3390/tropicalmed7120402

**Published:** 2022-11-28

**Authors:** Hafiz Muhammad Zeeshan, Aqsa Rubab, Hilda Dhlakama, Ropo Ebenezer Ogunsakin, Moses Okpeku

**Affiliations:** 1 Computer Science Department, National College of Business Administration and Economics, Lahore 54660, Pakistan; 2 School of Biomedical Sciences, University of Hong Kong, Hong Kong 999077, China; 3 Department of Statistics, University of Johannesburg, Johannesburg 2092, South Africa; 4 Discipline of Public Health Medicine, School of Nursing & Public Health, College of Health Sciences, University of KwaZulu-Natal, Durban 4000, South Africa; 5 Discipline of Genetics, School of Life Sciences, College of Agriculture, Engineering and Sciences, University of KwaZulu- Natal, Durban 4041, South Africa

**Keywords:** monkeypox, scopus, science mapping, bibliometric analysis, VOS viewer

## Abstract

Monkeypox is a zoonotic viral disease that has recently emerged as another global infection disease. A double-stranded enveloped deoxyribonucleic acid virus the cause of this disease. Since monkeypox is an evolving field of study with a growing interest in public health, it is crucial to study the scientific trend and research activities. This study provides an essential insight into the research response to scientific trends of monkeypox using the bibliometric analysis technique. A literature search for published articles on LSD from 2001 to 2021 was conducted in Scopus on 24 July 2022. Visualization analysis was performed using R statistical software. The growth and trend of documents, country-level distribution of publications and collaborations, and the relationship between authors and co-authors were analyzed. Findings revealed a significant increase in the research conducted, mainly from the United States (US). The top 12 institutions published papers on the monkeypox virus, accounting for 33.09 percent of the articles. The US was the most productive nation, producing 275 documents (54.34%), or one-third of all publications in this sector worldwide. Centers for Disease Control and Prevention in Georgia in the United States were the organization that produced the most (365 publications). The Journal of Virology garnered the most citations, with an h-index of 18. In the last year, there has been an increase in the publication of monkeypox virus-related studies. The importance of the monkeypox virus highlights the necessity for continued research to help international health organizations identify areas that require prompt action to implement suitable solutions. This study also provides scaling-up analysis, evidence dissemination on the monkeypox virus, emerging hotspots, and perceptive remarks on the technological advances in this field.

## 1. Introduction

Viruses are responsible for many medically necessary emerging and reemerging infections and various human and animal infectious disorders. They pose a far more significant threat to global public health today than a century ago [1]. Further, since they can spread swiftly, viruses significantly contribute to the morbidity and mortality of infectious diseases globally [2,3]. Human monkeypox is a zoonotic viral disease caused by the monkeypox virus (MPV), capable of transmission between animals and humans and secondary transmission between humans [4,5]. It is a member of the Orthopoxvirus (OPV) genus of the *Poxviridae* family [4]. Previously, MPV was first identified as a non-human pathogenic primate, that is, until human monkey smallpox was reported in the Democratic Republic of the Congo. Clinical expressions of human smallpox were like smallpox. For instance, in 2003, the United States (US) experienced an outbreak of human monkey smallpox, the first confirmed disease incidence outside of Africa [6,7]. Since then, the cumulative number of cases of monkeypox has steadily increased, and research on this topic has been increasingly conducted. However, there is still no comprehensive report to help researchers gain insight and understand global research trends in MPV. 

Bibliometric analysis is a tool for obtaining information and developmental trends about scientific activity in a specific field to collect quantifiable, reproducible, and objective data. This statistical technique is vital owing to its distinct advantages and wide range of applications in various research fields. This scientific approach has been used in multiple disciplines of study, including health sciences and engineering [8,9,10], to establish different patterns or trends. In this study, the growth rate of publications and the characteristics of research activities (keywords) were calculated, publication patterns (countries and journals) and research hotspot tendencies (citation). Despite the methodological limitations of bibliometric studies, they remain valuable tools for assessing the scientific importance of a selected discipline [8] since the method provides insight into the growth, size, and distribution of scientific literature in the field of interest within a specified time frame [8]. However, there are few bibliometric studies in the field of MPV research. Bibliometric analysis is a novel scientific method that integrates mathematical and statistical approaches with data visualization to determine the overall knowledge structure, development trends, and research priorities in a specific field [6,10].

Additionally, several studies have been published on MPV worldwide [11,12,13,14,15,16,17,18,19], and findings from such studies have helped to obtain vital information about state-of-the-art research and determine gaps in it. For young researchers to determine research trends and hotspots, the current study employed a method of statistical analysis called bibliometrics to assess the significant evolution of knowledge trends based on published research [6,8,10]. Therefore, in the current study, we conducted a bibliometric analysis of literature on monkeypox published using the Scopus database. The Scopus database was preferred because it provides better coverage of journal abstracts and citations than other databases such as Web of Science and PubMed [20,21]. The analyses comprised the number of annual publications, country contributions, international collaborations, institutions, journals, and authors. Thus, the primary objectives of this study are to identify the highly cited articles in research, their citation rate, and author counts per article. In addition, the study analyzes the significant contributing journals’ characteristics, reveals productive authors, shows the most contributing countries and attributes of the most cited papers, and reveals the life cycle of the most cited articles in MPV research. We hope this study can provide a new perspective and reference for future research on MPV.

## 2. Materials and Methods

A bibliometric technique condenses the most illuminating outcomes of a collection of bibliographic articles. It emphasizes author and institutional performance and the ways in which that affects the scientific output [22,23]. For bibliometric analysis, this study uses Bibliometrix, an R tool with a web-based interface, and Biblioshiny [8,24,25]. A comprehensive literature search was performed using the Scopus database for published articles on MPV. Bibliographic information on publicly available data were retrieved and downloaded from Scopus. The search was conducted on 24 July 2022. We searched for titles, abstracts, and keywords in the Scopus database. “Monkeypox” or “monkeypox virus” were the search terms. The Scopus database was chosen because it provides more extensive coverage of journal abstracts and citations compared to other databases such as PubMed and Web of Science [26,27]. Extracting this data did not involve interaction with human subjects or animals. As a result, no ethical issues were associated with using this data, and no ethics board approval was required. The search was performed daily to avoid bias caused by daily database updates. In the present study, only original articles published in English language were included. The search retrieved 383 items in English that met the inclusion criteria. After filtering using the inclusion and exclusion criteria, 501 publications were found. Three hundred and ninety-nine (399) documents were ultimately included in the final bibliometric analysis (Table 1). 

Furthermore, after filtering using the inclusion and exclusion criteria, 501 publications were found, as shown in Table 1. The paper lists were exported to CSV files, and the biblioshiny interface was used to create the Excel line charts and visualization maps. Therefore, the present study followed the five stages that authorize quantitative and qualitative investigation for bibliometric analysis [10,28]. These five stages regarding the bibliometric analysis procedure are the following: (1) Identification of study field and time of era; (2) selection of information sources; (3) search criteria; (4) identification (record identification); and (5) record analysis. Therefore, the main goal of this study remained to relate studies from multiple decades, nations, and journals to identical research standards. For the performance analysis, the number of annual publications, citations, authors, institutions, countries, and journal sources in Scopus was retrieved, summarized, and visualized using the bibliometrix R package, which has essential statistical and science-mapping analytical algorithms. A biblioshiny is included in more recent versions of the bibliometrix R-package to assist users without coding in conducting bibliometric analysis. VOSviewer also achieved a cluster by analyzing the frequency of the exact keywords appearing within the different documents. Accordingly, various nodes in a map represent elements, including a country, institution, or keywords. The size of the nodes reflects the number of publications or frequencies of keywords or authors; the more significant the node, the greater the number of publications or frequency [10]. The thickness of lines connecting pairs of nodes reflects the strength of co-occurrences or collaborations. The color of nodes and lines represents different clusters [10]. 

## 3. Results

### 3.1. Bibliometric Analysis of Published Articles on MPV 

A total of 501 scientific documents were found on the topic of MPV using the Scopus database between 2001 and 2021, comprising 399 (79.64%) original research papers and 108 (20.36%) review papers. According to these observations, original research articles were more prevalent. The average number of citations per document was 11.1 in one group, and the average number of citations per document was 32.44 in another; the average year from the date of publication was 4.84, and there were 18,320 references. The document contents included more than 4546 citation identifications (ID) and 767 author keywords (DE). The findings revealed 2067 authors, of whom 52 published their as sole authors and 22 published with multiple authors. Seven articles were included in the yearly publishing flow from 2001 detailing the number of documents, and the highest annual publication from 2005 detailed the number of records (44 articles). The number of papers grew in 2005, indicating rapid research interest in MPV. Surprisingly, the number of documents increased significantly to 26 in 2020, showing significant findings in the same year. Due to the critical discoveries on MPV, the number of records increased steadily during 2005, reaching its most considerable level ever (44). Analyzing the number of times MPV documents were cited allowed us to determine the average number of citations per year. 

### 3.2. Most Local Citation and Local Impact of Sources

Table 2 displays the findings of the top 20 sources that concentrated on MPV articles and had the most local impact, together with the top 20 sources that had the greatest number of significant local citations based on the cited references. The h factor indicates high citations. The most commonly mentioned local sources were the Journal of Virology, which had 1829 articles, and Virology, which had 1128 articles. The third and fourth journals of general virology and emerging infectious diseases had 582 and 580 papers, respectively. Molecules, Virology (817), Journal of General Virology (755), Emerging Infectious Disease (1194), and Journal of Virology (1294) were among the publications with a high citation rate according to the report (552). Notably, the journal ranked 17th with 17 local citations (h-index: 9, m-index: 15, and TC: 235, respectively). Virology and the Journal of General Virology placed third and fourth for the local source effect, with TCs of 817 and 755 and corresponding h indices of 14 and 8. The Journal of General Virology and Emerging Infectious Disease kept the top two positions regarding geographical effect, while Nature fell from fourth to seventh. “PLoS ONE”, formerly known as PLoS ONE, an open-access, peer-reviewed scientific publication published by the Public Library of Science (PLoS) since 2006 was another close source of effect. The journal publishes topics that span disciplines and several academic fields.

### 3.3. Most Local and Global Cited Documents

This section lists the works that have received the most local and international citations. The top 20 articles from 2001 to 2021 that received the most local and global mentions are listed in Table 3 and Table 4. A report’s “local citation” count refers to the number of sources it used to support the data analysis from other articles that focused on the topic. A piece of content’s “global citation” is the total number of citations across the database. It measures the impact of a piece of writing, which frequently receives most of its critical citations from works on unrelated themes. Surprisingly, the effect of the study on MPV research and other existing research projects increases with the local citation (LC). Meanwhile, the number of citations does not necessarily reflect the quality of an article, but it is a quantifier of its impact and visibility in the research area. The article published by GALDIERO S et al. [29] titled “Silver nanoparticles as Potential Antiviral Agents” was the most frequently globally cited article, with a TC of 552 and a value of 46 total citations per year. The next most globally cited article was the paper published by Lloyd-Smith JO et al. [30], “Epidemie dynamics at the human-animal interface”, published in 2009, with a TC of 401 and a value of 28.64 total citations. The 2004 publication by Reed KD et al. came third [31] titled “The finding of Monkeypox in Humans in the Western Hemisphere” and appeared in the New England Journal of Medicine, gaining 397 citations. A study of 2510 contacts of 214 individuals with human monkeypox was conducted in Zaire (now Democratic Republic of Congo) between 1980 and 1984. Among the contacts of the 130 primary cases, 62 secondary cases and 22 co-primary human monkeypox cases were discovered, while 14 more individuals with no clinical symptoms had positive serological results [32].

The article “NonHuman Primates have protected against smallpox virus or monkeypox virus Challenges by the Antiviral drag” also contributed the most papers among the 20 most frequently referenced publications, demonstrating its supremacy. According to local citation data, the research by Galdiero S, Wolfe ND, Gubser C, Yang G, Earl PL, Hooper JW, Hutin YJ, Rogers JV, Reed KD, and Likos AM was among the 20 most often cited papers both globally and locally. Regarding local citations, the study by Reed KD et al. was notable for coming in first. The article was given a total of 397 worldwide and 127 local citations. Likos AM wrote the second most cited local article. The third most cited article was written by Chen N. A noteworthy discovery was that Reed KD’s published work garnered more local citations than global citations.

### 3.4. Word Cloud of the Most Popular Keywords

**Figure 1:**
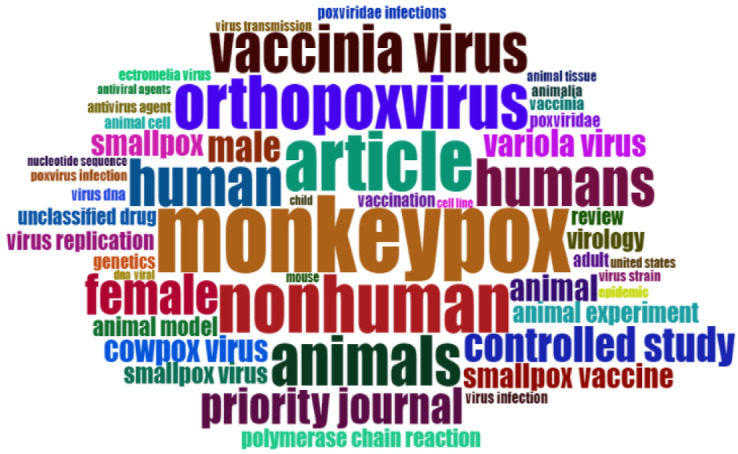
Word cloud of author keywords frequency during 2001–2021 (the size of keywords represents the frequency of occurrence).

The results of the word-cloud analysis utilizing the authors’ keywords are displayed in Figure 1. A word cloud (Figure 1) was built to visualize the counted frequency of more than 100 times the author keywords. The word cloud presents a visualization of the words that appeared most frequently in the papers on MPV research. The most common word was “monkeypox,” the second most common word was “orthopoxvirus,” and the third most common word was “animal.” The word cloud displays words in various sizes according to the number of times they appear. This word cloud provides more importance (reflected by text size) to author keywords that appear more frequently and, thus, serves as a visualization method to reveal the known research focus and trend in MPV research.

### 3.5. Analyzing Co-Occurrence Networks with Keywords and Keywords Plus

**Figure 2:**
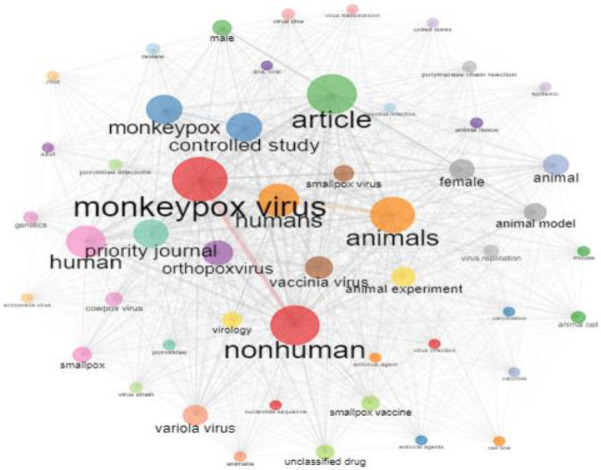
Co-occurrence network visualization for MPV research: keywords.

Figure 2 shows the MPV study’s network of keyword co-occurrences. The co-occurrences of writers’ keywords fall into two categories. The size of the nodes in the keyword-co-occurrences network, which determines the frequency of two phrases appearing together, indicates the quantity of author keyword co-occurrence. Consequently, the importance of the keywords rises as node size climbs. The node colors reflect different clusters, while the lines between the nodes reveal phrase frequency. Links between nodes define their connections to one another. 

### 3.6. Diffusion of Author Keywords

**Figure 3:**
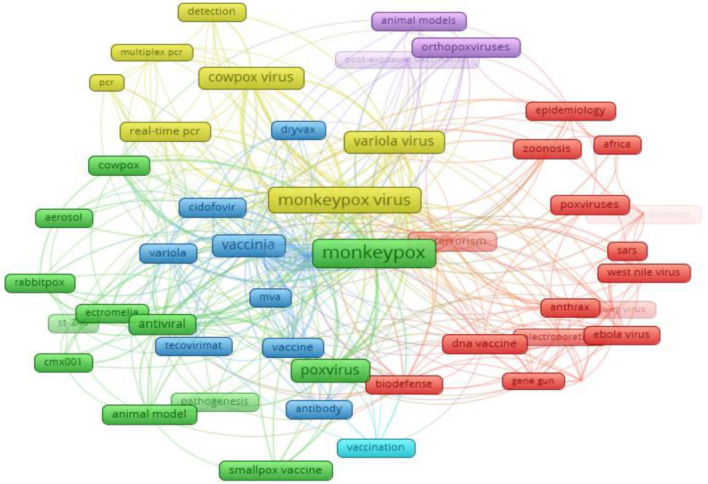
Top 50 author keywords found in MPV-related documents. Green, Red, Blue and Pink are the first four clusters; Red, the second; Yellow, the third; Blue, the fourth; and Pink, the fifth.

The bibliometric analysis of the author keywords from the present study period discovered 767 author keywords. The author keywords in articles that referred to MPV were evaluated, and the top 50 author keywords were used and clustered from 2001 to 2022 (Figure 3). The node and word size depict the node weight, while the spacing between them signifies the intensity of the association between them. The lines between the keywords emphasize that they appeared simultaneously. The thicker the line, the more the co-occurrence. Nodes with the same color are grouped. The top four most frequently used keywords were ‘monkeypox’, ‘monkeypox virus’, ‘cowpox virus’, and ‘variola viru’s’, which agreed with the research trend (Figure 3).

Furthermore, for the keyword diffusion plus, the top 50 most popular keywords and the co-word networks were investigated and shown using the biblioshiny interface. According to the findings of the keywords-plus survey, the top four most frequently used terms were “monkeypox”, “monkeypox virus”, “orthopoxvirus”, and “smallpox virus”. The author keywords and the keywords plus had some similarities. The top 50 most popular searches included words such as “vaccination”, “poxvirus”, “monkeypox”, “monkeypox virus”, “smallpox virus”, “orthopoxvirus”, “cowpox virus”, “real-time pcr”, “antiviral”, “animal model”, and “pox virus”, which are like the author keyword findings (Figure 3).

### 3.7. Collaboration by Academic

**Figure 4:**
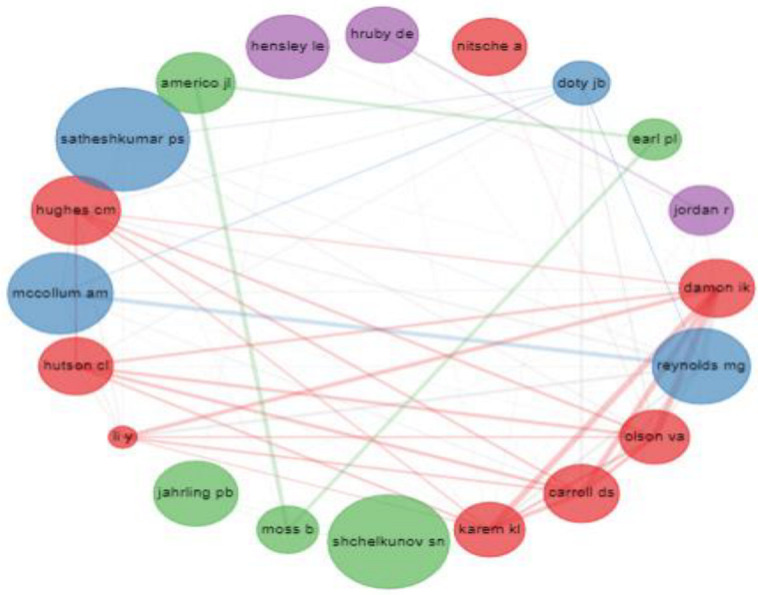
Graphical representation of the collaboration between the authors.

Collaboration amongst academic authors usually promotes knowledge and exchange, thereby widening the topic under investigation. At several levels, there were observable cooperation relationships in MPV research (Figure 4). To find out which author contributed the most, we evaluated them based on their total number of citations. Upon this, we observed that Damon I.K. and Karem K.L. came first regarding co-citations. The two authors made special recognized for their excellent contributions to MPV research. The author-collaboration map demonstrates how the writers cooperated in their scientific research. The authors are represented by the rectangle/node, while the number of articles is represented by the size of the circle/node. The colors reflect the clusters, while the lines demonstrate the writers’ abilities to operate as a team. The degree of connectivity between the nodes, in particular, reveals how frequently people collaborate [10].

### 3.8. Top Most Cited Countries and Global Collaboration

**Figure 5:**
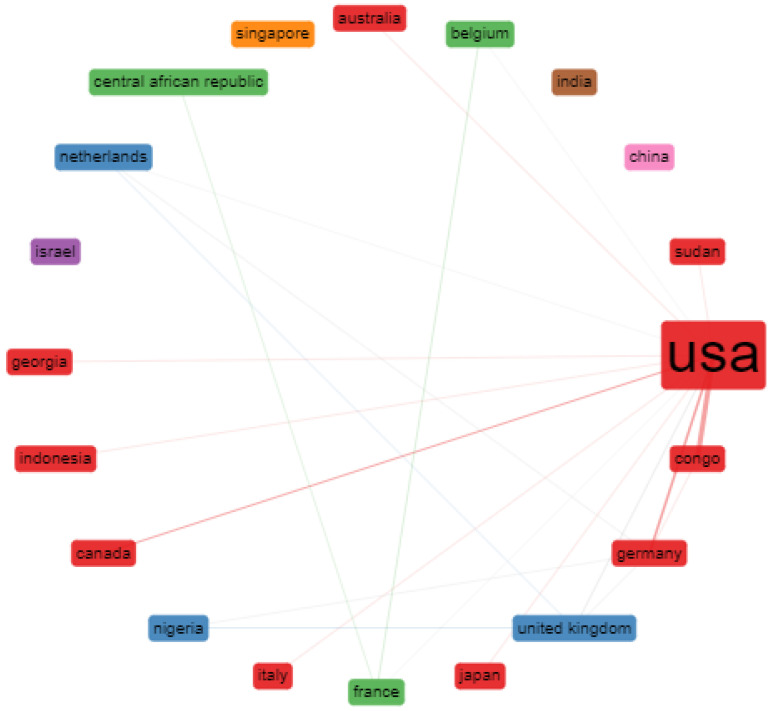
Visualization of international collaboration.

Table 5 shows the top 20 most cited countries with their total and average article citations. Interestingly, the four nations with the most citations were the United States, Germany, the United Kingdom, and Italy, with average article citations of 39.31, 32.27, 44.67.78, and 74.63, respectively. The four colors on the map suggest that research trends are becoming more varied. The country collaboration network of MPV-related articles is shown in Figure 5. Furthermore, evaluating inter-institutional collaborations is a helpful way to assess the level of partnership—the countries’ exchange of country-to-country engagement with the leading nations in this area. The critical nodes represent the significant countries, while the connections between the nodes represent institutional linkages. The distance between nodes and the linkage strength shows the cooperation of the nations with one another.

Additionally, the thicker the link between the countries, the stronger the collaborative relationship, and vice versa. The USA, Georgia, Indonesia, Italy, and Congo had the closest ties in this collaborative network. The United States led the most prominent group outside its immediate geographic region. It worked along with neighbors such as Canada, as well as with nations in Asia and Europe. The United Kingdom led the second group with China and Japan. It might also be considered one of the hubs for the entire network in terms of cross-border cooperation. The third group, led by Germany, had many traits of that of the United States. Germany maintained close ties with Israel and developed productive alliances with European nations such as Belgium and the Netherlands. The fourth group led by France collaborated with India, Sudan, and the Central African Republic. 

### 3.9. Bibliometric Analysis of Institution Collaborations and Most Important Affiliations 

**Figure 6:**
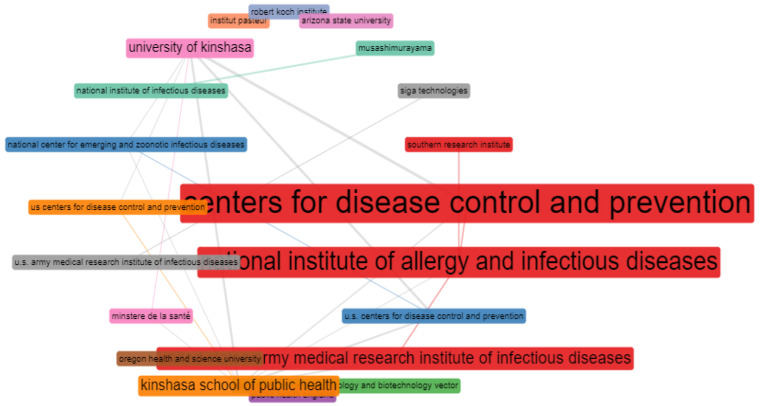
Visualization of institutions collaborations.

Figure 6 shows the network collaboration of MPV-related articles. The results obtained from the analysis show that the United States Center for Disease Control and Prevention had the highest collaboration network, followed by the National institute of allergy and infectious diseases, etc. In contrast, Arizona State University has no collaboration network. Individual nodes denote the different universities/institutes, and each node’s diameter denotes the institution’s collaboration strength with other institutions; the lines signify the collaboration networks or pathways between each institution, while the thickness of the lines signifies the collaboration strength. 

## 4. Discussion

The drive of this paper was to examine the tracking knowledge evolution and systematic evaluation of research trends in MPV through a bibliometric approach. We observed a substantial growth of research output in the field, with most papers published in recent years. The global pandemic prompted the biomedical community to explore and develop antiviral therapies after being evaluated by several virology laboratories. The MPV consequently resulted in many research publications. Therefore, a thorough evaluation of the state of MPV research is required to direct future research objectives, mainly through partnerships between various academic researchers in numerous fields. As a result, the current study’s aim was to look at the publication trends, prolific writers, journals, and countries in the MPV field. This study provides a global bibliometric analysis of MPV research. Therefore, the results of this work may influence a further theoretical investigation. According to past research, the number of publications published over time may be a good measure of production and advancement [62,63]. The rise in publications on relevant subjects indicated that MPV received significant and increasing attention from scientists. 

Additionally, between 2016 and 2018, the bulk of the documents included in the MPV research had average TCs of 15, 8, and 16 per year; however, the specific number of citations dropped to 8 in 2017 and dramatically increased to 44 in 2022. The most significant regional and global citations for MPV research came from the Journal of Virology, then Emerging Infectious Disease. As well, our research showed changes in the number of publications on MPV during the study time. This observation had close relation to the findings of past bibliometric literature assessments [10,63]. It was evident that there had been an overall increase in publishing in 2021 when the number of articles on MPV for that year was displayed. It is hoped that MPV will release content more quickly in the future. 

The analysis also provides a national-level overview of the way MPV research has changed over the past 21 years in countries across several continents. Germany and the United States were particularly active compared to the African nations. Additionally, the findings indicate that American articles dominate all other scientific publications. Although the United States considerably outperformed the competition, Germany and the United Kingdom are the two nations that dominate MPV research. According to our research, the USA partnered more frequently than any other nation.

In addition to collaborating with nations in Asia and Europe, the USA also worked with neighboring countries. This demonstrates that these nations made significant financial, material, and human investments in scientific research. It is not surprising that they have become global leaders in MPV research since other bibliometric studies have also produced identical results [63]. The results also show that due to frequent academic exchanges between the two countries, scholars from the United States, India, China, and the United Kingdom collaborated most closely. In addition, international researchers kept cooperating within the scope of the global network. However, regarding citations and h-index, Germany was the most powerful nation. Additionally, the most influential countries had different publication rates, with the USA having the highest number of publications (*n* = 10,809), followed by Germany (*n* = 839) and the United Kingdom (*n* = 670). The implication is that international publications could boost a country’s stature and influence in MPV research.

Moreover, the findings also demonstrated that a network of cooperation linked most countries, as shown by the lines on the network map. The large h-index number, which showed that the topic had many readers and citations, illustrated the MPV research’s global reach. The most-cited academic articles on the MPV epidemic are another example of how widely known the MPV epidemic was. The data furthermore showed that the Centers for Disease Control and Prevention in the United States played crucial roles in MPV research when it came to the institutions and organizations that were most pertinent. I.K. Damon and M.G. Reynolds were the authors who had the most significant impact on the MPV research. The results of the keyword co-occurrence analysis showed various areas of interest in research for scientists. The words “monkeypox” and “monkeypox virus” were most frequently used.

As previously discussed, there was a fair collaboration regarding MPV as indicated by the circle size in the author keyword co-occurrence networks; indicated by the many articles contained the author’s keyword. However, the strength of the correlation revealed how closely the two terms were discovered. Clusters of nodes that shared many co-occurrences were found for each bibliometric network and colored [8,10]. As a result, this study carefully assessed the advancement of MPV research by utilizing bibliometric analytic methods. However, more interdisciplinary, multi-institutional, and multinational research collaborations are required to advance this field of study, according to the current state of MPV research collaborations worldwide. Likewise, the author keywords and keyword co-occurrence analysis results revealed various research areas for individual research scientists. More general keywords simplify the search for articles, improving the chances of detecting more quotes. The advantage of keywords is that they help researchers identify the domain more quickly, efficiently, and effectively, and they can help reveal the structure of domain knowledge. Thus, based on our findings, the top frequently occurring keywords using the author keywords show dominant words such as ‘monkeypox’, ‘monkeypox virus’, ‘cowpox virus’, and ‘variola virus’.

Further, the university collaboration analysis showed that the United States Center for Disease Control and Prevention had the most collaborative or network strength. Looking at the prominent research institutions that generated most of the articles on MPV, it is noteworthy that these organizations have a long history of research and have high scores in many areas. For instance, the United States institutions appear as highly productive institutions in other bibliometric studies [64]. The findings indicated that the United States Center for Disease Control and Prevention had played a significant role in MPV research. 

## 5. Study Limitations

The current study is not without limitation. First, the literature data sources for this study are limited to the Scopus database, which may not be enough for comprehensive assessment of MPV. Although, eligible papers in journals not included in Scopus were obviously excluded in the present study, we obtained documents from high-quality international journals that are the most influential source of scientific communication in the field. Other databases, such as PubMed and Web of Science, could be explored in future investigations. Secondly, we may have missed some articles that do not use instructive keywords in the title because we did not look for the terminology used in abstracts. We did not review reference lists of eligible articles to identify any potentially missing papers.

Furthermore, we did not include grey literature, and we did not include articles published in languages other than English. As such, we may have missed relevant documents published in conferences proceeding and gazettes. Finally, we assessed the scientific research literature exclusively and identified trends concerning scientific discovery likely to impact new research efforts.

## 6. Conclusions

We conducted keyword analysis in combination with title search to gain insight into the studied exposures and used methodologies. Based on the three hundred and ninety-nine publications, this bibliometric study provides a comprehensive overview of research in MPV and evaluates the literature information at different years, country collaborations, institutions, authors, and journals and analyzes the theme development and future research hotspots. This finding would assist in generating evidence-based reports, evaluations, and visualizations of research outputs on MPV research. Our study provides basic information about research in this field and identifies potential collaborators for interested researchers. Various topics and keywords were popularly used in this theme and could potentially be further developed. In addition, some of the most productive journals and authors can also be used as references for researchers working on MPV. Thus, it is essential that the management targeting human monkeypox deserve further attention. 

## Figures and Tables

**Table 1:** Primary Information on MPV Research Publications (2001–2021).

Description	Results
**MAIN INFORMATION ABOUT DATA**	
Timespan	2001:2021
Sources (journals, books, etc.)	203
Documents	501
Annual growth Rate %	4.84
Document average age	11.1
Average citations per doc	32.44
References	18,320
**DOCUMENT CONTENTS**	
Keywords plus (ID)	4546
Author keywords (DE)	767
**AUTHORS**	
Authors	2067
Authors of single-authored docs	43
**AUTHORS COLLABORATION**	
Single-authored documents	52
Co-Authors per document	7.35
International co-authorships %	22.75
**DOCUMENT TYPES**	
Article	399
Review	102

**Table 2:** Top 20 local sources with the most local impact.

Journal	Most Significant Local Source Impact				Maximum Local Citation	Documents
	h_Index	g_Index	m_Index	TC.		
Journal of Virology	18	31	0.947	1294	Journal of Virology	1829
Emerging Infectious Disease	16	19	0.727	1194	Virology	1128
PLoS ONE	14	23	0.875	538	Journal of General Virology	582
Virology	14	20	0.7	817	Emerging Infectious Diseases	580
Vaccine	11	16	0.688	496	Vaccine	412
American Journal of Tropical Medicine and Hygiene	10	14	0.556	526	The Journal of Infectious Diseases	383
Antimicrobial Agents and Chemotherapy	9	9	0.429	388	Nature	329
Viruses	9	15	0.692	235	Science	269
Antiviral Research	8	12	0.381	441	PLoS ONE	257
Journal of General Virology	8	9	0.421	755	The Lancet	243
Journal of Infectious Disease	8	10	0.421	356	New England Journal of Medicine	222
Journal of Virology Methods	7	9	0.5	125	Bulletin of the World Health Organization	217
Journal of Clinical Microbiology	6	8	0.35	201	Antiviral Research	206
PLOS Neglected Tropical Diseases	6	7	0.286	426	Antimicrob Agents Chemother	198
Clinical Infectious Diseases	5	6	0.75	99	American Journal of Tropical Medicine and Hygiene	195
Journal of Medical Virology	5	5	0.278	222	Journal of Clinical Microbiology	172
PLoS Pathogens	5	7	0.278	123	Clinical Infectious Diseases	171
Journal of Clinical Virology	4	7	0.385	219	Journal of Infectious Disease	156
Virus Research	4	4	0.235	118	Journal of Immunology	154
Molecular and Cellular Proteomics	4	4	0.308	57	Cell	128

**Table 3:** The top 20 articles that were mentioned the most on a global scale.

Documents	Digital Object Identifier	Total Citation	Total Citation per Year	Normalized Total Citation
Galdiero S [29], 2011, MOLECULES	10.3390/molecules16108894	552	46	12.77
Lloyd-Smith JO [30], 2009, SCIENCE	10.1126/science.1177345	401	28.64	8.57
Reed KD [31], 2004, NEW ENGL J MED	10.1056/NEJMoa032299	397	20.89	5.19
Wolfe ND [33], 2005, EMERG INFECT DIS	10.3201/eid1112.040789	371	20.61	6.34
Rogers JV [34], 2008, NANOSCALE RES LETT	10.1007/s11671-008-9128-2	315	21	8.1
Gubser C [35], 2004, J GEN VIROL	10.1099/vir.0.19565-0	284	14.95	3.71
Earl PL [36], 2004, NATURE	10.1038/nature02331	273	14.37	3.57
Yang G [37], 2005, J VIROL	10.1128/JVI.79.20.13139-13149.2005	232	12.89	3.96
Hurin YJ [38], 2001, EMERGING INFECT DIS	10.3201/eid0703.017311	229	10.41	3
Di Giulio DB [14], 2004, LANCET INFECT DIS	10.1016/S1473-3099(03)00856-9	214	11.26	2.8
Edghill-Smith Y [39], 2005, NAT MED	10.1038/nm1261	211	11.72	3.61
Hooper JW [40], 2004, J VIROL	10.1128/JVI.78.9.4433-4443.2004	186	9.79	2.43
Likos AM [41], 2005, J GEN VIROL	10.1099/vir.0.81215-0	183	10.17	3.13
Baker RO [42], 2003, ANTIVIRAL RES	10.1016/S0166-3542(02)00196-1	153	7.65	4.49
Zaucha GM [43], 2001, LAB INVEST	10.1038/labinvest.3780373	142	6.45	1.86
Chen N [44], 2005, VIROLOGY	10.1016/j.virol.2005.05.030	139	7.72	2.38
Parker S [45], 2007, FUTURE MICROBIOL	10.2217/17460913.2.1.17	125	7.81	3.27
Guarner J [46], 2004, EMERG INFECT DIS	10.3201/eid1003.030878	125	6.58	1.63
Learned LA [47], 2005, AM J TROP MED HYG	10.4269/ajtmh.2005.73.428	116	6.44	1.98
Stittelaar KJ [48], 2006, NATURE	10.1038/nature04295	111	6.53	2.44

**Table 4:** The top 20 locally cited documents.

Document	Digital Object Identifier	Year	LC.	GC.	LC/GC Ratio (%)
Reed KD [31], 2004, NEW ENGL J MED	10.1056/NEJMoa032299	2004	127	397	31.99
Likos AM [41], 2005, J GEN VIROL	10.1099/vir.0.81215-0	2005	85	183	46.45
Chen N [44], 2005, VIROLOGY	10.1016/j.virol.2005.05.030	2005	67	139	48.2
Zaucha GM [43], 2001, LAB INVEST	10.1038/labinvest.3780373	2001	67	142	47.18
Hutin YJ [49], 2001, EMERGING INFECT DIS	10.3201/eid0703.017311	2001	64	229	27.95
Dl Glulio DB [50], 2004, LANCET INFECT DIS	10.1016/S1473-3099(03)00856-9	2004	58	214	27.1
Earl PL [51], 2004, NATURE	10.1038/nature02331	2004	51	273	18.68
Meyer H [52], 2002, J CLIN MICROBIOL	10.1128/JCM.40.8.2919-2921.2002	2002	51	105	48.57
Parker S [45], 2007, FUTURE MICROBIOL	10.2217/17460913.2.1.17	2007	49	125	39.2
Huhn GD [53], 2005, CLIN INFECT DIS	10.1086/498115	2005	47	87	54.02
Huston CL [54], 2007, AM J TROP MED HYG	10.4269/ajtmh.2007.76.757	2007	46	87	52.87
Yang G [55], 2005, J VIROL	10.1128/JVI.79.20.13139-13149.2005	2005	45	232	19.4
Hooper JW [56], 2004, J VIROL	10.1128/JVI.78.9.4433-4443.2004	2004	43	186	23.12
Reynolds MG [57], 2006, J INFECT DIS	10.1086/505880	2006	42	91	46.15
LI Y [58], 2006, J CLIN VIROL	10.1016/j.jcv.2006.03.012	2006	42	84	50
Huston CL [59], 2009, J GEN VIROL	10.1099/vir.0.005108-0	2009	39	66	59.09
Guarner J [46], 2004, EMERG INFECT DIS	10.3201/eid1003.030878	2004	38	125	30.4
Edghill-Smith Y [39], 2005, NAT MED	10.1038/nm1261	2005	37	211	17.54
Shchelkunov SN [60], 2001, FEBS LETT	10.1016/S0014-5793(01)03144-1	2001	36	103	34.95
Huggins J [61], 2009, ANTIMICROB AGENTS CHEMOTHER	10.1128/AAC.00021-09	2009	31	87	35.63

**Table 5:** Countries with the highest total and average article citations.

Country	Total Citations	Article Average Citations
USA	10,809	39.31
Germany	839	32.27
United Kingdom	670	44.67
Italy	597	74.63
Australia	383	54.71
Netherlands	281	56.2
France	162	16.2
Israel	149	29.8
Japan	132	18.86
Canada	114	28.5
Spain	112	18.67
Greece	81	81
India	63	21
Nigeria	60	20
Finland	54	27
Belgium	46	23
Switzerland	44	44
Ireland	41	41
Kenya	34	34
Austria	33	11

## Data Availability

Raw and processed data are available to the first and second authors upon request.

## References

[1] Howard C.R., Fletcher N.F. (2012). Emerging virus diseases: Can we ever expect the unexpected?. Emerg. Microbes Infect..

[2] Jones K.E., Patel N.G., Levy M.A., Storeygard A., Balk D., Gittleman J.L., Daszak P. (2008). Global trends in emerging infectious diseases. Nature.

[3] Woolhouse M.E.J., Gowtage-Sequeria S. (2005). Host range and emerging and reemerging pathogens. Emerg. Infect. Dis.

[4] Xiang Y., White A. (2022). Monkeypox virus emerges from the shadow of its more infamous cousin: Family biology matters. Emerg. Microbes Infect..

[5] Hraib M., Jouni S., Albitar M.M., Alaidi S., Alshehabi Z. (2022). The outbreak of monkeypox 2022: An overview. Ann. Med. Surg..

[6] Cheng K., Zhou Y., Wu H. (2022). Bibliometric analysis of global research trends on monkeypox: Are we ready to face this challenge?. J. Med. Virol..

[7] Petersen E., Abubakar I., Ihekweazu C., Heymann D., Ntoumi F., Blumberg L., Asogun D., Mukonka V., Lule S.A., Bates M. (2019). Monkeypox—Enhancing public health preparedness for an emerging lethal human zoonotic epidemic threat in the wake of the smallpox post-eradication era. Int. J. Infect. Dis..

[8] Ogunsakin R.E., Ebenezer O., Jordaan M.A., Shapi M., Ginindza T.G. (2022). Mapping Scientific Productivity Trends and Hotspots in Remdesivir Research Publications: A Bibliometric Study from 2016 to 2021. Int. J. Environ. Res. Public Health.

[9] Tan H., Li J., He M., Li J., Zhi D., Qin F., Zhang C. (2021). Global evolution of research on green energy and environmental technologies:A bibliometric study. J Environ. Manag..

[10] Ogunsakin R.E., Ebenezer O., Ginindza T.G. (2022). A Bibliometric Analysis of the Literature on Norovirus Disease from 1991–2021. Int. J. Environ. Res. Public Health.

[11] Heymann D.L., Szczeniowski M., Esteves K. (1998). Re-emergence of monkeypox in Africa: A review of the past six years. Br. Med. Bull..

[12] Jezek Z., Grab B., Szczeniowski M.V., Paluku K.M., Mutombo M. (1988). Human monkeypox: Secondary attack rates. Bull. World Health Organ..

[13] Stanford M.M., McFadden G., Karupiah G., Chaudhri G. (2007). Immunopathogenesis of poxvirus infections: Forecasting the impending storm. Immunol. Cell Biol..

[14] Grant R., Nguyen L.-B.L., Breban R. (2020). Modelling human-to-human transmission of monkeypox. To cite this version: HAL Id: Hal-03287459 Modelling human-to-human transmission of monkeypox. Bull. World Health Organ..

[15] Nalca A., Rimoin A.W., Bavari S., Whitehouse C.A. (2005). Reemergence of monkeypox: Prevalence, diagnostics, and countermeasures. Clin. Infect. Dis..

[16] MacNeil A., Reynolds M.G., Braden Z., Carroll D.S., Bostik V., Karem K., Smith S.K., Davidson W., Li Y., Moundeli A. (2009). Transmission of atypical varicella-zoster virus infections involving palm and sole manifestations in an area with monkeypox endemicity. Clin. Infect. Dis..

[17] Brown K., Leggat P.A. (2016). Human monkeypox: Current state of knowledge and implications for the future. Trop. Med. Infect. Dis..

[18] Patrono L.V., Pléh K., Samuni L., Ulrich M., Röthemeier C., Sachse A., Muschter S., Nitsche A., Couacy-Hymann E., Boesch C. (2020). Monkeypox virus emergence in wild chimpanzees reveals distinct clinical outcomes and viral diversity. Nat. Microbiol..

[19] Okyay R.A., Bayrak E., Kaya E., Şahin A.R., Koçyiğit B.F., Taşdoğan A.M., Avci A., Sümbül H.E. (2022). Another Epidemic in the Shadow of Covid 19 Pandemic: A Review of Monkeypox. Eurasian J. Med. Oncol..

[20] Burnham J.F. (2006). Scopus database: A review. Biomed. Digit. Libr..

[21] Pranckutė R. (2021). Web of science (Wos) and scopus: The titans of bibliographic information in today’s academic world. Publications.

[22] Tuppurainen E.S.M., Lamien C.E., Diallo A. (2021). Capripox (Lumpy Skin Disease, Sheep Pox, and Goat Pox). Veterinary Vaccines: Principles and Applications.

[23] Rahman M. (2020). Outbreaks of Lumpy Skin Disease of Cattle in Bangladesh: What to Know and What to Do. SSRN Electron. J..

[24] Sajovic I., Podgornik B.B. (2022). Bibliometric Analysis of Visualizations in Computer Graphics: A Study. SAGE Open.

[25] Koo M. (2021). Systemic lupus erythematosus research: A bibliometric analysis over a 50-year period. Int. J. Environ. Res. Public Health.

[26] Yang W., Zhang J., Ma R. (2020). The prediction of infectious diseases: A bibliometric analysis. Int. J. Environ. Res. Public Health.

[27] Zhang X., Estoque R.C., Xie H., Murayama Y., Ranagalage M. (2019). Bibliometric analysis of highly cited articles on ecosystem services. PLoS ONE.

[28] Churiyah M., Sholikhan S., Filianti F. (2022). Mobile learning uses in vocational high school: A bibliometric analysis. World J. Educ. Technol. Curr. Issues.

[29] Galdiero S., Falanga A., Vitiello M., Cantisani M., Marra V., Galdiero M. (2011). Silver nanoparticles as potential antiviral agents. Molecules.

[30] Lloyd-Smith J.O., George D., Pepin K.M., Pitzer V.E., Pulliam J.R., Dobson A.P., Hudson P.J., Grenfell B.T. (2009). Epidemie dynamics at the human-animal interface. Science.

[31] Reed K.D., Melski J.W., Graham M.B., Regnery R.L., Sotir M.J., Wegner M.V., Kazmierczak J.J., Stratman E.J., Li Y., Fairley J.A. (2004). The Detection of Monkeypox in Humans in the Western Hemisphere From the Departments of Pathology. N. Engl. J. Med..

[32] Jezek Z., Marennikova S.S., Mutumbo M., Nakano J.H., Paluku K.M., Szczeniowski M. (1986). Human monkeypox: A study of 2510 contacts of 214 patients. J. Infect. Dis..

[33] Wolfe N.D., Daszak P., Kilpatrick A.M., Burke D.S. (2005). Bushmeat hunting, deforestation, and prediction of zoonotic disease emergence. Emerg. Infect. Dis..

[34] Rogers J.V., Parkinson C.V., Choi Y.W., Speshock J.L., Hussain S.M. (2008). A preliminary assessment of silver nanoparticle inhibition of monkeypox virus plaque formation. Nanoscale Res. Lett..

[35] Gubser C., Hué S., Kellam P., Smith G.L. (2004). Poxvirus genomes: A phylogenetic analysis. J. Gen. Virol..

[36] Earl P.L., Americo J.L., Wyatt L.S., Eller L.A., Whitbeck J.C., Cohen G.H., Eisenberg R.J., Hartmann C.J., Jackson D.L., Kulesh D.A. (2004). Immunogenicity of a highly attenuated MVA smallpox vaccine and protection against monkeypox. Nature.

[37] Yang G., Pevear D.C., Davies M.H., Collett M.S., Bailey T., Rippen S., Barone L., Burns C., Rhodes G., Tohan S. (2005). An Orally Bioavailable Antipoxvirus Compound (ST-246) Inhibits Extracellular Virus Formation and Protects Mice from Lethal Orthopoxvirus Challenge. J. Virol..

[38] Yinka-Ogunleye A., Aruna O., Ogoina D., Aworabhi N., Eteng W., Badaru S., Mohammed A., Agenyi J., Etebu E.N., Numbere T. (2018). Reemergence of human monkeypox in Nigeria, 2017. Emerg. Infect. Dis..

[39] Edghill-Smith Y., Golding H., Manischewitz J., King L.R., Scott D., Bray M., Nalca A., Hooper J.W., Whitehouse C.A., Schmitz J.E. (2005). Smallpox vaccine–induced antibodies are necessary and sufficient for protection against monkeypox virus. Nat. Med..

[40] Hooper J.W., Thompson E., Wilhelmsen C., Zimmerman M., Ichou M.A., Steffen S.E., Schmaljohn C.S., Schmaljohn A.L., Jahrling P.B. (2004). Smallpox DNA Vaccine Protects Nonhuman Primates against Lethal Monkeypox. J. Virol..

[41] Likos A.M., Sammons S.A., Olson V.A., Frace A.M., Li Y., Olsen-Rasmussen M., Davidson W., Galloway R., Khristova M.L., Reynolds M.G. (2005). A tale of two clades: Monkeypox viruses. J. Gen. Virol..

[42] Baker R.O., Bray M., Huggins J.W. (2003). Potential antiviral therapeutics for smallpox, monkeypox and other orthopoxvirus infections. Antivir. Res..

[43] Zaucha G.M., Jahrling P.B., Geisbert T.W., Swearengen J.R., Hensley L. (2001). The pathology of experimental aerosolized monkeypox virus infection in cynomolgus monkeys (*Macaca fascicularis*). Lab. Investig..

[44] Chen N., Li G., Liszewski M.K., Atkinson J.P., Jahrling P.B., Feng Z., Schriewer J., Buck C., Wang C., Lefkowitz E.J. (2005). Virulence differences between monkeypox virus isolates from West Africa and the Congo basin. Virology.

[45] Parker S., Nuara A., Buller R.M.L., Schultz D.A. (2007). Human monkeypox: An emerging zoonotic disease. Future Microbiol..

[46] Guarner J., Johnson B.J., Paddock C.D., Shieh W.J., Goldsmith C.S., Reynolds M.G., Damon I.K., Regnery R.L., Zaki S.R., the Veterinary Monkeypox Virus Working Group (2004). Monkeypox Transmission and Pathogenesis in Prairie Dogs. Emerg. Infect. Dis..

[47] ten Have H. (2022). Emerging Infectious Diseases. Adv. Glob. Bioeth..

[48] Stittelaar K.J., Neyts J., Naesens L., Van Amerongen G., Van Lavieren R.F., Holý A., de Clercq E., Niesters H.G.M., Fries E., Maas C. (2006). Antiviral treatment is more effective than smallpox vaccination upon lethal monkeypox virus infection. Nature.

[49] Hutin Y.J.F., Williams R.J., Malfait P., Pebody R., Loparev V.N., Ropp S.L., Rodriguez M., Knight J.C., Tshioko F.K., Khan A.S. (2001). Outbreak of Human Monkeypox, Democratic Republic of Congo, 1996 to 1997. Emerg. Infect. Dis..

[50] Di Giulio D.B., Eckburg P.B. (2004). Human monkeypox: An emerging zoonosis. Lancet Infect. Dis..

[51] Townsend M.B., Keckler M.S., Patel N., Davies D.H., Felgner P., Damon I.K., Karem K.L. (2013). Humoral immunity to smallpox vaccines and monkeypox virus challenge: Proteomic assessment and clinical correlations. J. Virol..

[52] Meyer H., Perrichot M., Stemmler M., Emmerich P., Schmitz H., Varaine F., Shungu R., Tshioko F., Formenty P. (2002). Outbreaks of Disease Suspected of Being Due to Human Monkeypox Virus Infection in the Democratic Republic of Congo in 2001. J. Clin. Microbiol..

[53] Huhn G.D., Bauer A.M., Yorita K., Graham M.B., Sejvar J., Likos A., Damon I.K., Reynolds M.G., Kuehnert M.J. (2005). Clinical characteristics of human monkeypox, and risk factors for severe disease. Clin. Infect. Dis..

[54] Hutson C.L., Lee K.N., Abel J., Carroll D.S., Montgomery J.M., Olson V.A., Li Y., Davidson W., Hughes C., Dillon M. (2007). Monkeypox zoonotic associations: Insights from laboratory evaluation of animals associated with the multi-state US outbreak. Am. J. Trop. Med. Hyg..

[55] Jordan R., Leeds J.M., Tyavanagimatt S., Hruby D.E. (2010). Development of ST-246® for treatment of poxvirus infections. Viruses.

[56] Buchman G.W., Cohen M.E., Xiao Y., Richardson-Harman N., Silvera P., DeTolla L.J., Davis H.L., Eisenberg R.J., Cohen G.H., Isaacs S.N. (2010). A protein-based smallpox vaccine protects non-human primates from a lethal monkeypox virus challenge. Vaccine.

[57] Reynolds M.G., Yorita K.L., Kuehnert M.J., Davidson W.B., Huhn G.D., Holman R.C., Damon I.K. (2006). Clinical manifestations of human monkeypox influenced by route of infection. J. Infect. Dis..

[58] Li Y., Olson V.A., Laue T., Laker M.T., Damon I.K. (2006). Detection of monkeypox virus with real-time PCR assays. J. Clin. Virol..

[59] Hutson C.L., Olson V.A., Carroll D.D., Abel J.A., Hughes C.M., Braden Z.H., Weiss S., Self J., Osorio J.E., Hudson P.N. (2009). A prairie dog animal model of systemic orthopoxvirus disease using west African and Congo Basin strains of Monkeypox virus. J. Gen. Virol..

[60] Shchelkunov S.N., Totmenin A.V., Babkin I.V., Safronov P.F., Ryazankina O.I., Petrov N.A., Gutorov V.V., Uvarova E.A., Mikheev M.V., Sisler J.R. (2001). Human monkeypox and smallpox viruses: Genomic comparison. FEBS Lett..

[61] Huggins J., Goff A., Hensley L., Mucker E., Shamblin J., Wlazlowski C., Johnson W., Chapman J., Larsen T., Twenhafel N. (2009). Non-human primates are protected from smallpox virus or monkeypox virus challenges by the antiviral drug ST-246. Antimicrob. Agents Chemother..

[62] Falagas M.E., Papastamataki P.A., Bliziotis I.A. (2006). A bibliometric analysis of research productivity in parasitology by different world regions during a 9-year period (1995–2003). BMC Infect. Dis..

[63] Garg K.C., Rao M.K.D. (1988). Bibliometric analysis of scientific productivity: A case study of an Indian physics laboratory. Scientometrics.

[64] Fan J., Gao Y., Zhao N., Dai R., Zhang H., Feng X., Shi G., Tian J., Chen C., Hambly B.D. (2020). Bibliometric analysis on COVID-19: A comparison of research between English and Chinese studies. Front. Public Health.

